# In Vivo Radioprotective Potential of Newly Synthesized Azomethine and Styrylquinoline Derivatives and a Natural Polyphenol: A Preliminary Study

**DOI:** 10.3390/life12030346

**Published:** 2022-02-26

**Authors:** Nevena Nikolova, Donika Ivanova, Zvezdelina Yaneva

**Affiliations:** 1 Ecology with Radioecology Unit, Department of General Livestock Breeding, Faculty of Veterinary Medicine, Students Campus, Trakia University, 6000 Stara Zagora, Bulgaria; 2 Chemistry Unit, Department of Pharmacology, Animal Physiology, Biochemistry and Chemistry, Faculty of Veterinary Medicine, Students Campus, Trakia University, 6000 Stara Zagora, Bulgaria; donika.ivanova@trakia-uni.bg (D.I.); zvezdelina.yaneva@trakia-uni.bg (Z.Y.)

**Keywords:** synthetic azomethines, styrylquinolinic salts, silymarin, gamma irradiation, radioprotection

## Abstract

The aim of the present study was to investigate the radioprotective activity of silymarin, a plant substance with hepatoprotective activity, of four newly synthesized structural derivatives of anthranilic acid azomethines, and alkyl-2-styrylquinolinic acid, as well as to establish and assess the influence of the solvent type and bioactive compound dose on the in vivo radioprotective potential of the natural and novel synthetic compounds. Male Wistar strain rats weighing 110–120 g were used for the in vivo experiments. Fifteen minutes after i.p. injection of the compounds, the experimental animals were irradiated by 8 Gy. Results indicate that the compound 2-{[(3,5-dihydro-2-hydroxyphenyl)methylen] amino}-benzoic acid in a dose of 60 mg/kg body weight exhibited the highest radioprotective effect, whereas the natural extract silymarin did not manifest radioprotective potential, even in high doses.

## 1. Introduction

Ionizing radiations are deleterious in nature and cause direct and indirect DNA damage due to a cascade of events leading to the generation of free radicals. Therefore, to prevent free-radical-associated diseases, the most common strategy is to quench the generated free radicals with efficient and non-toxic synthetic and/or natural agents [1]. 

The search for new effective radioprotective agents is carried out both by studying the chemical structure of natural biologically active compounds and obtaining their derivatives, as well as by synthesizing radioprotectors based on experimentally established molecular structure/radioprotective effect dependencies and activity relations. Screening compounds as potential radioprotectors is a mandatory step in the accumulation of such dependencies, but the lack of international standardized systems for pre-selection and comprehensive characterization of candidate protectors causes serious difficulties. Uncertainty is created both from the omission of effective compounds due to “false-negative” results and from the high material and intellectual costs of “false-positive” screening results. 

Amino benzoic acids have been extensively studied due to their broad range of biological activities, including antifungal, antibacterial, antimalarial, antiproliferative, anti-inflammatory, antiviral, antipyretic, and antioxidant properties [2,3,4]. Amino benzoic acid derivatives have been synthesized and evaluated for their anticholinesterase potential [5]. According to a study by Krátký et al. [6], various anthranilic acid hybrid compounds have been reported as potential antimicrobial agents, exhibiting improved radical scavenging properties and enhanced toxicity for cancer cells. Thus, chemical modifications could provide viable concepts for modulation of amino benzoic acid properties to obtain physiologically active compounds with antimicrobial and cytotoxic potential [6]. 

Azomethine compounds possess an azomethine group (>C=N–) as a result of the reaction between the carbonyl group of an aldehyde or ketone and the nucleophilic primary amine through addition–elimination reactions. The presence of a substituent attached to an azomethine is reported to affect the antioxidant activity of the derivatives [7]. In a study by Geronikaki et al. [8], azomethine derivatives were evaluated for their anti-inflammatory and antioxidant activity, as some had already been tested as antiviral, antibacterial, antimycotic, and antimycobacterial agents. Other studies have observed the importance of azomethines for their antibacterial, antifungal, antiproliferative, and antipyretic properties [9]. According to Chigurupati et al. [9] azomethines with aryl substituents are more stable and readily synthesized, whereas those containing alkyl substituents are relatively unstable. Azomethines of aliphatic aldehydes are usually unstable and readily polymerizable, whereas those of aromatic aldehydes with effective conjugation are more stable.

Quinoline scaffold has attracted immense attention in the field of medicinal chemistry, as it is one of the 35 key building blocks of a number of natural bioactive compounds. The quinoline ring is endowed with various activities, such as antituberculosis, antimalarial, anti-inflammatory, anticancer, antibiotic, antihypertensive, and anti-HIV activities [10]. Styrylquinolines have earned scientific interest due to the discovery of their biological activities, profound mechanisms of action, and their potential as drug candidates in clinical trials [11]. Mirzaei et al. [12] established that quinolines possessing N-(4-benzoyl phenyl) and N-(4-phenoxyphenyl) exhibited strong cytotoxic activity against four human cancer cells. 

Natural polyphenols have displayed radioprotective efficiency during various investigations, outlining the possibility of their administration at high doses with lower toxicity. Detoxification of free radicals, modulating inflammatory responses, DNA repair, stimulation of hematopoietic recovery, and immune functions constitute the major mechanisms for radiation protection. According to Adnan et al. [13], epicatechin, epigallocatechin-3-gallate, apigenin, caffeic acid phenylethyl ester, and silibinin provide cytoprotection combined with the suppression of many pro-inflammatory cytokines, owing to their free-radical scavenging, antioxidant, and anti-inflammatory properties. Nagpal et al. [1] established antimutagenic and antioxidant activity of tea polyphenols and β-carotene against γ-radiation-induced mutation and oxidative stress in the larvae and adult flies of *D. melanogaster*. It was documented that the radioprotective activity of curcumin, resveratrol, quercetin, gallic acid, and rutin are primarily regulated by direct or indirect decline in cellular stress. Based on modern scientific literature, polyphenols have been outlined as potential radioprotective candidates. However, extensive investigation is still required to better understand the protection mechanisms. 

The wide range of scientifically proven biological activities of anthranilic acid azomethine and styrylquinoline derivatives and natural bioactive compounds on the one hand and the necessity for the development of test and control screening methodologies for rapid and safe initial detection of the radioprotective activity of synthetic and natural compounds on the other hand provoked the current investigations.

The aim of the present study was to investigate the radioprotective activity of silymarin, plant substance with hepatoprotective activity, of four newly synthesized structural derivatives of anthranilic acid azomethines and alkyl-2-styrylquinolinic acid, as well as to establish and assess the influence of the solvent type and bioactive compound dose on the in vivo radioprotective potential of the natural and novel synthetic compounds.

## 2. Materials and Methods

### 2.1. Chemical Reagents

The following chemical reagents were used in the organic synthesis reactions: 3,5-dichlorosalicylaldehyde (Cl_2_C_6_H_2_(OH)CHO, 99%; CAS No. 90-60-8), anthranilic acid (2-(NH_2_)C_6_H_4_COOH; for synthesis; CAS No. 118-92-3) 2,3-dihydroxybenzaldehyde ((HO)_2_C_6_H_3_CHO, 97%, CAS No. 24677-78-9), and ethanol (C_2_H_5_OH, p.a., absolute, ≥99.8%, CAS No. 64-17-5), 2,4,6-trimethoxy benzaldehyde ((CH_3_O)_3_C_6_H_2_CHO, 98%, CAS No. 830-79-5), supplied by Sigma-Aldrich (St. Louis, MA, USA); and 1,2-dimethylquinolinium iodide (C_11_H_12_IN, CAS No: 876-87-9), supplied by BioSynth CarboSynth (Staad, Switzerland).

### 2.2. Experimental Instrumentation

^1^H (600.1 and 250.1 MHz) and ^13^C (150.9 MHz) NMR spectra were acquired on Bruker AVANCE AV600 II+ and Bruker DRX 250 NMR spectrometers (Bruker Optik GmbH, Ettlingen, Germany). Chemical shifts are expressed as δ-values (ppm) relative to TMS as an internal standard, using CDCl_3_ and DMSO-d_6_ as solvents. The following contractions indicate signal multiplicity and assignment: s (singlet), d (doublet), t (triplet), q (quartet), m (multiplet), brs (broad singlet), and dd (doublet of doublets).

Elemental analyses were performed on a Perkin Elmer 2400 CHNSO analyzer (Perkin Elmer, Norwalk, CT, USA). 

IR spectra were obtained with the potassium bromide (KBr) disc technique in the range of 400–4000 cm^−1^ using a TENSOR 37 Bruker FTIR spectrometer (Bruker Optik GmbH, Ettlingen, Germany).

UV–Vis spectra were scanned with a Hach Lange DR 5000 UV–Vis spectrophotometer (Hach Lange, D-40549 Düsseldorf, Germany) supplied with 10 mm quartz cells. All spectra were recorded with a 2 nm slit width, 900 nm min^−1^ scan speed, and very high smoothing.

The newly synthesized compounds were characterized by their melting points, measured on a Wagner Munz Koffler hot bench system (Wagner & Munz GmbH, Munich, Germany), without correction and by elemental analysis.

### 2.3. Synthesis of New Azomethyne Derivatives and Styrylquinolinic Acid Salts 

**Scheme 1:**
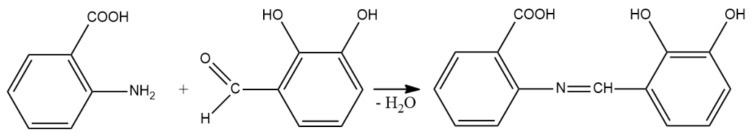
Nucleophilic addition–elimination reaction for the synthesis of AZM1.

**Scheme 2:**
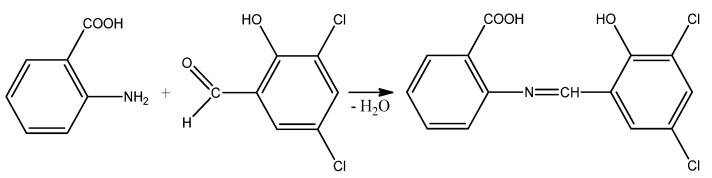
Nucleophilic addition–elimination reaction for the synthesis of AZM2.

The following newly synthesized azomethine derivatives were used in the present study: 2-{[(2.3-dihydroxyphenyl)methylene]amino}benzoic acid (AZM1) and 2-{[(3,5-dichloro-2-hydroxiphenil)methylene]amino} benzoic acid (AZM2) were synthesized by the nucleophilic addition reaction between 2-aminobenzoic acid (anthranilic acid) with two different substituted aromatic aldehydes by a method described by Lashin et al., with some modifications [14] (Scheme 1 and Scheme 2).

#### 2.3.1. Synthesis of 2-{[(2.3-Dihydroxyphenyl)methylene]amino} Benzoic Acid (AZM1)

Anthranilic acid (0.69 g; 0.005 mol) dissolved in 30 cm^3^ hot distilled water was mixed with 0.69 g (0.005 mol) 2,3-dihydroxibenzaldehyde dissolved in 10 cm^3^ hot distilled water. The reaction mixture was heated to reflux for 30 min. Activated carbon was added to the boiling solution. The mixture was filtered while hot. The solid reaction product, a red crystalline substance with a melting point of 222–223 °C, was obtained after cooling, followed by recrystallization from ethanol, with a 90% theoretical yield.

Elemental analyses: C, H, N (M = 257.26 g/mol)

Theoretical: C% 65.36; H% 4.31; N% 5.44;

Experimental: C% 65.27; H% 4.26; N% 5.36.

**Figure 1:**
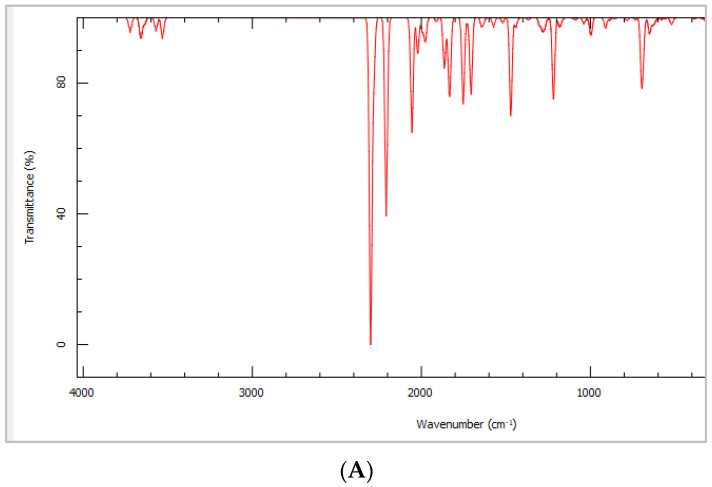

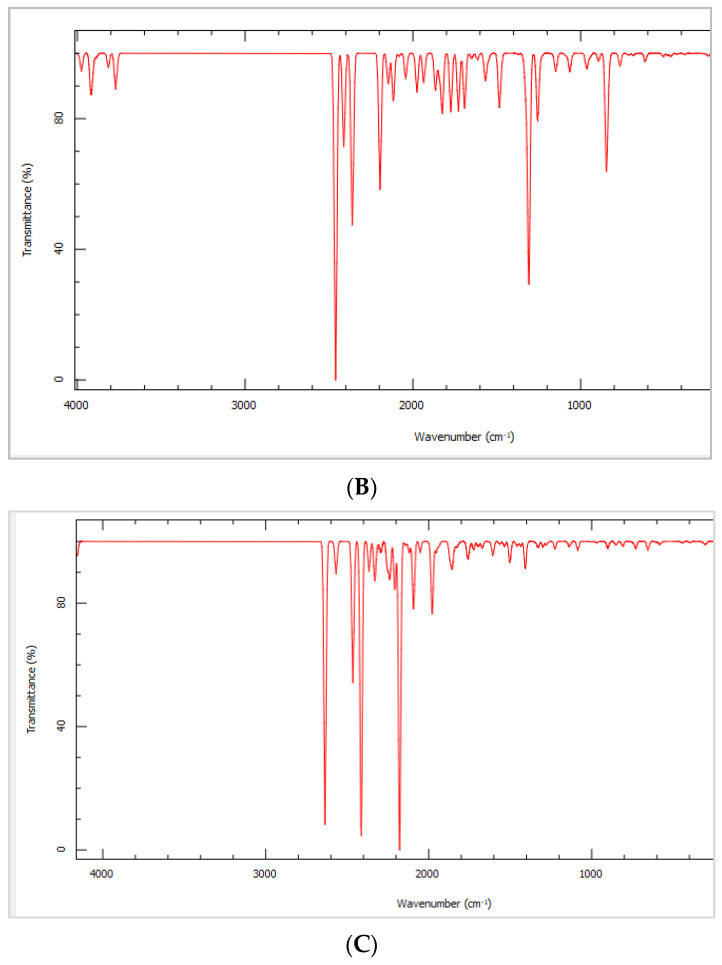
IR spectra of (**A**). AZM1, (**B**). AZM2, and (**C**). SQI.

^13^C NMR (CDCl_3_) δ 166.5 (COOH), 163.9 (C=NR), 160.0 (C-N), 151.7 (C-OH), 146.1 (C-OH), 135.2 (CH), 128.2 (CH), 126.5 (C-H), 124.7 (CH), 124.1 (CH), 122.8 (CH), 119.9 (C), 119.6 (CH), 116.3 (C); ^1^H NMR (DMSO): at δ 11.00 [s, 1H, COOH], 5.35 [s, 2H, C-OH], 8.21–7.66 [m, 4H, Ar-H], 7.22–6.81 [m, 3H’, Ar-H), 8.39 (s, 1H, HC=N); IR: 2255 (N=C); 2165, 2015 (C=C); 1796, 1718 (C=O); 1442 (O-H); 1194 (O-H) (Figure 1A); UV–Vis: λ = 330 nm.

#### 2.3.2. Synthesis of 2-{[(3,5-Dichloro-2-hydroxyphenyl)methylene]amino} Benzoic Acid (AZM2)

Anthranilic acid (0.69 g; 0.005 mol) and 3,5-dichlorosalicyl aldehyde (0.95 g; 0.005 mol) were dissolved in 25 cm^3^ absolute EtOH. The mixture was heated to reflux for 3 h. The solid reaction product, a red crystalline substance with a melting point of 208–210 °C, was obtained after cooling, followed by recrystallization from ethanol, with an 80 % theoretical yield.

Elemental analyses: C, H, N (M = 309.43 g/mol)

Theoretical: C% 54.34, H% 2.93, N% 4.52;

Experimental: C% 54.86, H% 3.21, N% 4.31.

^13^C NMR (CDCl_3_) δ 166.5 (COOH), 160.0 (C=NR), 157.9 (C-OH), 153.9 (C-N), 135.2 (CH), 134.1 (CH), 128.7 (CH), 128.4 (C-Cl), 128.2 (CH), 126.9 (C-Cl), 126,5 (CH), 124.1 (CH), 121.3 (C), 116.3 (C); ^1^H NMR (DMSO): at δ 11.00 [s, 1H, COOH], 5.35 [s, 1H, C-OH], 8.21–7.66 [m, 4H, Ar-H], 7.53–7.48 [m, 2H’, Ar-H), 8.87 (s, 1H, HC=N); IR: 3459 (O-H); 2257 (N=C); 2166, 2014 (C=C); 1674 (C=N); 1201, 1152 (C-O); 777 (C-Cl) (Figure 1B); UV–Vis: λ = 240 nm.

DMSO was chosen as a solvent because of its ability to solubilize anthranilic acid azomethines, as well as its relatively low toxicity (LD_50_ = 12.2 g/kg) and the absence of cytogenic and teratogenic activity.

An aqueous solution of AZM2 sodium salt (AZM2*) obtained by a reaction with NaOH was studied in doses of 60 mg/kg and 200 mg/kg. 

#### 2.3.3. Synthesis of a New Alkyl-2-Styrylquinolinic Acid Salt 

**Scheme 3:**
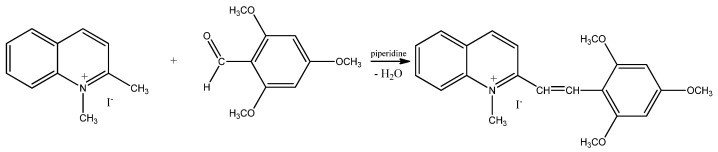
Synthesis reaction of SQI.

The compound N-methyl-2-(2,4,6-trimethoxy-styryl) quinoline iodide (SQI) was synthesized by a condensation reaction between 2,4,6-trimethoxy benzaldehyde and 1,2-dimethylquinolinium iodide according to the method of Varbanova and Chervenkov [15] (Scheme 3). We investigated the compound as a representative of the synthetic alkyl-2-styrylquinoline acid salt class. In the present study, aqueous solutions of SQI were allied in two doses lower than the toxic dose (LD = 2.38 mg/kg): minimum 0.3 mg/kg and maximum 1.8 mg/kg.

^13^C NMR (CDCl_3_) δ 160.6 (CO), 159.6 (CO), 159.4 ©, 134.7 (CH), 132.6 (C)133.12 (CH), 129.6 (CH), 128.2 (CH)127.8 (C), 127.0 (CH), 126.1 (CH), 119.3 (CH), 116.6 (CH), 101.7 (C), 90.9 (CH), 56.2 (CH_3_), 55.8 (OCH_3_), 34.0 (CH_3_); ^1^H NMR (DMSO): at δ 4.02 [s, 1H, CH_3_], 3.83 [s, 3H, C-OH], 6.09 [s, 2H, CH], 7.03, 7.83 [2s, 2H, HC=CH], 7.24, 8.08 [2s, 2H, Ar-H], 7.83–7.77 [m, 2H, CH, Ar-H), 8.08–7.57 [m, 3H, Ar-H); IR: 2465, 2416, 2330 (C-H); 2207 (C=N); 2178 (C=C); 2093; 1978 (C=C); 1407 (C-N) (Figure 1C); UV–Vis: λ = 210 nm.

### 2.4. Natural Flavonoid

The bioflavonoid silymarin (purified extract), a plant substance, is a mixture of flavonolignan isomers isolated from the seeds of *Silybum marianum* (milk thistle). It is the main component of the hepatoprotective drug “Carsil”, which was kindly provided by the manufacturer, Sopharma, Kazanlak, Bulgaria. The substance was used at factory purity without further purification according to OH-0277493-84. A solution of silymarin in aqueous alkaline medium (pH = 10) was used for the in vivo experiments with a minimum injection dose of 40 mg/kg body weight (5 times higher than the therapeutic dose) and a maximum injection dose of 160 mg/kg body weight (20 times higher than the therapeutic dose).

### 2.5. In Vivo Studies

The in vivo experiments were performed with male Wistar rats with a live weight of 110–120 g, divided into 13 groups (Table 1). Rats were acclimated to controlled laboratory conditions for two weeks and maintained on stock rodent diet and tap water ad libitum. Fifteen minutes after i.p. injection of the compounds, the experimental animals were irradiated by 8 Gy on a ROCUS-M gamma installation. All animal procedures were approved by the Animal Ethics Committee of the Faculty of Veterinary Medicine (animal testing license no. 233/09 April2019) and the Commission on Animal Ethics at the Bulgarian Food Safety Agency. The effect of the injection dose, solvent type, and organic compound type were studied. 

Group 1 (G1) rats were treated with the compound AZM1 dissolved in ethanol at a dose of 60 mg/kg body weight and irradiated with 8 Gy. 

Group 2 (G2) rats were treated with the compound AZM2 dissolved in ethanol at a dose of 60 mg/kg body weight and irradiated with 8 Gy. 

Group 3 (G3) rats were treated with the compound AZM2 dissolved in ethanol at a dose of 200 mg/kg body weight and irradiated with 8 Gy. 

Group 4 (G4) rats were treated with the compound AZM2 dissolved in DMSO at a dose of 60 mg/kg and irradiated with 8 Gy. 

Group 5 (G5) rats were treated with the compound AZM2* dissolved in distilled water at a dose of 60 mg/kg and irradiated with 8 Gy. 

Group 6 (G6) rats were treated with the compound AZM2* dissolved in water at a dose of 200 mg/kg body weight and irradiated with 8 Gy. 

Group 7 (G7) rats were treated with SQI dissolved in water at a dose of 0.3 mg/kg body weight and irradiated with 8 Gy. 

Group 8 (G8) rats were treated with SQL dissolved in distilled water at a dose of 1.8 mg/kg body weight and irradiated with 8 Gy. 

Group 9 (G9) rats were treated with silymarin dissolved in aqueous alkaline medium (pH = 10) at a dose of 40 mg/kg body weight and irradiated with 8 Gy. 

Group 10 (G10) rats were treated with silymarin dissolved in aqueous alkaline medium (pH = 10) at a dose of 160 mg/kg body weight and irradiated with 8 Gy. 

Group 11 (G11) (control 1) rats were treated with DMSO at a dose of 8000 mg/kg body weight and irradiated with 8 Gy. 

Group 12 (G12) (control 2) rats were not treated but were irradiated with 8 Gy. 

Group 13 (G13) (control 3) consisted of untreated and non-irradiated rats.

Irradiation was performed 15 min after intraperitoneal injection of the animals on a Gamma installation, ROCUS-M, at a rate of 0.02 Gy/s. The survival percentage was measured on the 10th, 15th, and 30th days of the experiment.

## 3. Results and Discussion

### 3.1. In Vivo Radioprotective Potential 

The experimental results of the radioprotective activity of the three synthetic compounds and the bioflavonoid, as well as the influence of the solvent type and injection dose, revealed total mortality of the rats from Groups 1, 5, 6, 9, 10, 11, and 12. The experimental data for Group 12 (control 2), consisting of only irradiated rats, proved that the applied dose of gamma radiation (8 Gy) was lethal to the animals; thus, the selected irradiation intensity was suitable for initial screening and evaluation of the radioprotective potential of the tested newly synthesized synthetic compounds, as well as the natural polyphenol (Table 1). Srinivas et al. [16] reported that the mean lethal dose, LD_50//30_, of Wistar rats exposed to whole-body X-ray irradiation through linear acceleration was 6.6 Gy, whereas animals exposed to 8 Gy died 11 days post irradiation.

**Figure 2:**
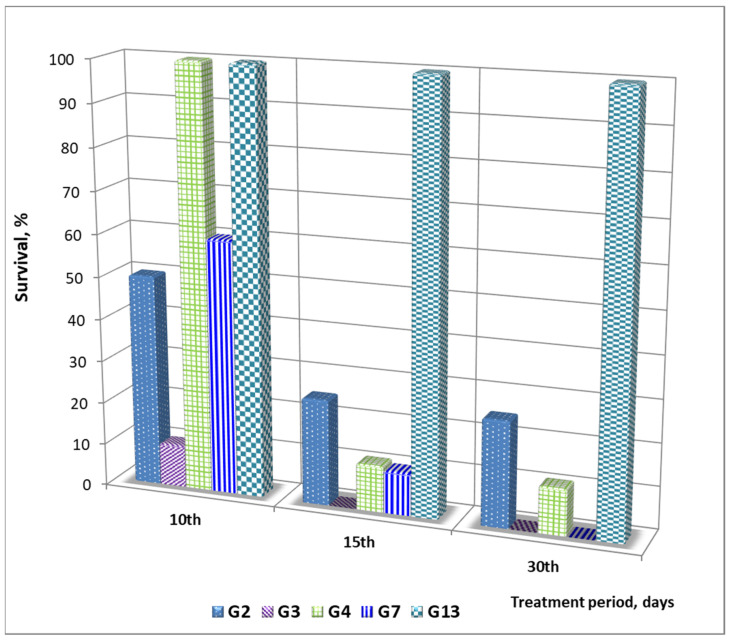
Comparative analyses of the in vivo radioprotective effect of AZM and SQI.

According to the results presented in Figure 2, the compound AZM2 exhibited a radioprotective effect at a dose of 60 mg/kg, which was influenced by the type of solvent used.

The rats injected with 60 mg/kg AZM2 and irradiated with 8 Gy displayed 50% survival with EtOH as solvent (G2) and 100% with DMSO (G4) solvent on the 10th day. However, the radioprotective activity of the compound in DMSO was significantly decreased on the 15th and 30th days. On the other hand, the activity of AZM2 in EtOH against γ-irradiation, although lower on the 10th day as compared to G4, was reduced to 25 % and remained constant until the 30th day. Obviously, the ethanol solution of AZM2 was able to provide more consistent radioprotection for a prolonged time period, whereas the DMSO solution was 100% efficient for a shorter period of irradiation exposure. Comparative analyses of the results for groups II and III revealed that the radioprotective effect of AZM2 was inversely proportional to its dose, which provides evidence for future studies with lower AZM2 doses. The Na salt of AZM2 (AZM2*) was characterized by improved water solubility, which is a presumption for increased radioprotective effect. The experimental results, however, proved the absence of any protective activity of the compound in both tested doses (60 mg/kg and 200 mg/kg). A probable explanation is the ionic structure of the salt, which changes the nature of the biological activity of the compound. 

Rats treated with N-methyl-2-styrylquinoline salt (SQI) at a dose of 0.3 mg/kg (G7) (Figure 2) showed 60% survival on the 10th day, followed by a rapid decrease to 10% on the 15th day and death of all animals in the group by the 30th day of irradiation. Similarly, to AZM2, the 6-fold increased injection dose of SQI resulted in total death of the treated rats in G8. 

The plant flavonoid silymarin dissolved in alkaline medium (pH = 10) in doses of 40 and 160 mg/kg (G9 and G10, respectively), 5–20 times the therapeutic dose, did not display radioprotective activity against the damage caused irradiation at 8 Gy despite its polyphenolic structure. Recently reported data (Table 2) prove the radioprotective effect of silymarin at gamma irradiation doses within the range of 1–7 Gy with plant extract injection doses of 50–100 mg/kg. In most of the investigations in the literature, silymarin was dissolved in water. The deviations in the effect of silymarin established by the present study as compared to literature data could be explained from two perspective. The first probable reason for the mortality of the rats is the high gamma radiation intensity, which was detrimental for the animals. The second explanation is the limited water solubility of the bioactive compounds in silymarin, which results in lower bioavailability of the natural extract and consequently lower therapeutic activity. Silymarin is composed primarily of silibinin and small amounts of other stereoisomers, such as silydianin and silychristin [16]. Hung et al. [16] studied the effect of the pH of buffers on skin permeation of silibinin within the range of pH 6–10.8. The authors reported that the ionization of the compound increased following an increase in pH. The observed saturated solubility of silibinin buffers at pH = 9.9 and pH = 10.8 were 190.53 and 565.10 μg/mL, respectively [17]. The latter results provoked us to apply alkaline water solution with pH = 10 as a solvent of silymarin in the present study. From another aspect, however, the increased solubility of the alkaline medium indicated that the ionic form of silibinin provided more hydrophilic molecular conditions. Considering that pH of aqueous vehicles is one of the major parameters influencing drug diffusivity [18,19], the lypophobicity of the ionized hydrophilic molecules is a perquisite of their limited mass transfer through biological membranes, resulting in lower radioprotective efficiency of the plant extract.

### 3.2. Influence of the Molecular Characteristics and Substituent Type on the Radioprotective Activity of AZM1, AZM2, AZM2*, and SQI

The mechanism of radioprotection is associated with the quenching of generated ROS by molecules exhibiting antioxidant and radical-scavenging potential, which is the main cause of irradiation-induced cellular damage. These activities are based on the ability of a neutral molecule to generate stable radicals. However, the mechanism of scavenging-radical generation depends on various factors, such as molecular structure, physicochemical properties, electronic effects, inter- and intramolecular interactions, environmental influence, etc. [28].

**Figure 3:**
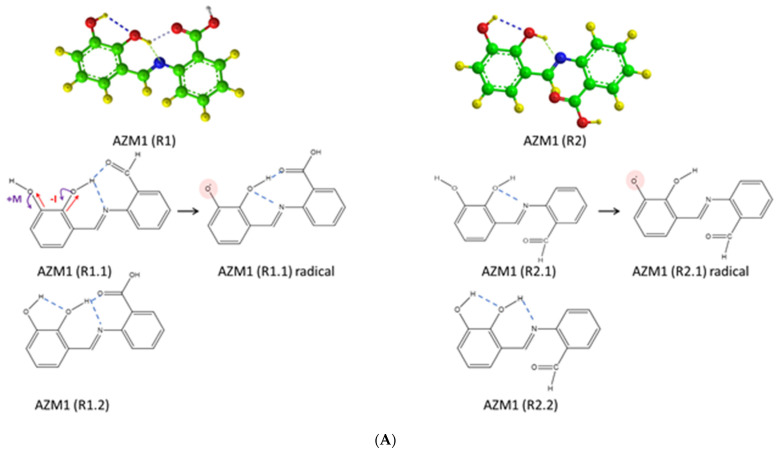

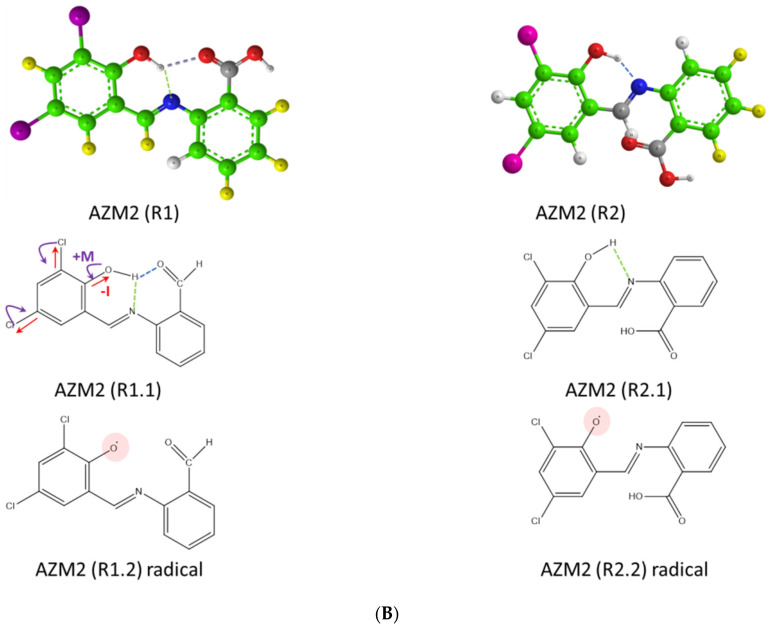
Inductive (I) and mesomeric (M) effects, intramolecular H-bonding (---), and radical formation possibilities of (**A**) AZM1 and (**B**) AZM2 rotamers.

The possible molecular mechanisms of the generation of scavenging radicals by the synthetic organic compounds AZM1 and AZM2 was assessed on the basis of substituent type and the electronic (inductive and mesomeric) effects within the molecules. The reason for the absence of radioprotective activity in AZM1 can be explained in terms of the molecular structure and effects of the phenolic derivatives. It has been reported that the presences of electron-withdrawing and electron-donating groups affects the bond-dissociation enthalpy of O-H and, consequently, the stabilization of the phenoxy radicals [29]. The two vicinal –OH groups in AZM1 molecules are electron-withdrawing substituents characterized by a negative inductive effect (-I), which increases intramolecular interactions, and resonance-donating substituents characterized by a positive mesomeric effect (+M) (Figure 3A) [30]. -I is a deactivating effect, and it is usually weaker than the mesomeric effect. Consequently, the –OH group is an activating group. AZM2 molecules contain one –OH group and two –Cl substituents (Figure 3B). Chlorine is also characterized by an -I effect, which results in electrons being withdrawn inductively, and an +M effect, which adds electron density back into the benzene ring. Due to the fact that for halides, the mesomeric effect is more dominant than the inductive effect, they are classified as mild deactivators. 

From another aspect, the formation of intramolecular hydrogen bonds is a prerequisite for weaker radical-scavenging activity due to blockage of the –OH groups, limiting the possibility of the formation of stable free radicals from the neutral molecule [28]. Therefore, we assessed the influence of intramolecular bonding in the possible rotamers of AZM1 and AZM2 (Figure 3A,B). Obviously, depending on the spatial orientation of the two vicinal –OH groups, the R1.1 and R2.1 rotamers could be transformed into radicals, as one of their –OH groups does not participate in H-bonding, whereas in the molecules of the R1.2 and R2.2 rotamers, there are two or three H-bonds, and both –OH groups are blocked. Similarly, R1.2 and R2.2 rotamers of AZM2 could form radicals, as they possess an unbonded –OH group, whereas the R1.1 and R2.1 rotamers could not exhibit radical scavenging activity. The latter observations lead to the conclusion that the higher radioprotective activity of AZM2 could be due to the presence of only one –OH group, which is more susceptible to homolytic deprotonation than vicinal AZM1 –OH groups.

**Figure 4:**
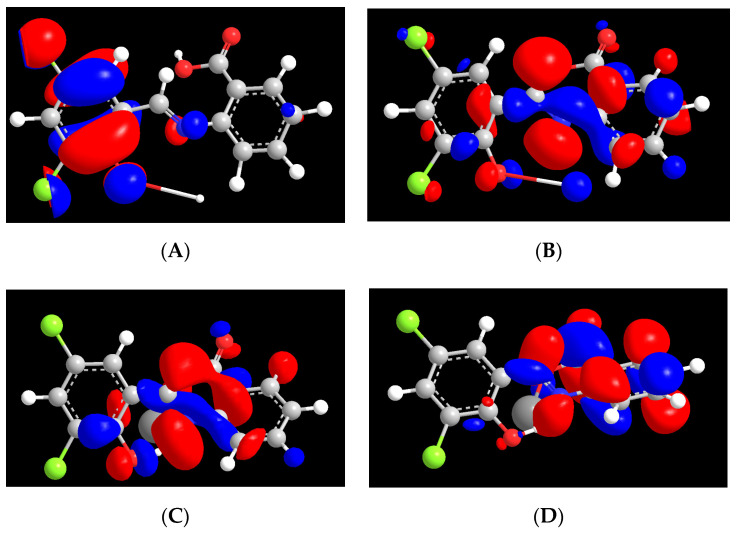
The HOMO/LUMO (highest occupied molecular orbital/lowest unoccupied molecular orbital) electron densities in the molecules of AZM2 and AZM2*. (**A**) HOMO of AZM2; (**B**) LUMO of AZM2; (**C**) HOMO of AZM2*; (**D**) LUMO of AZM2*.

According to Świsłocka et al. [31] the HOMO-LUMO energy gap could be applied as a useful descriptor of the chemical and biological activity of molecules. The smaller the gap value, the more chemically active the molecule is. Our comparative molecular modelling study of AZM2 and its sodium salt AZM2* (Figure 4), as well as the calculated molecular properties of HOMO and LUMO potentials (Table 3), revealed that the HOMO-LUMO energy gap of AZM2* surpassed that of AZM2 by 10 times, which is indicative of the lower biochemical activity of the salt. Consequently, the experimental results depicting unproductive protection of the aqueous solution of AZM2* were proven by the molecular modelling data. 

In agreement with the obtained results, the scientific literature contains reports of a variety of biological activities of salicylaldehyde derivatives containing one or more halo-atoms in the aromatic ring, such as antioxidant, antibacterial, and antifungal activity [32,33,34].

We observed the antiretroviral activity of several styrylquinoline derivatives that have been reported to be HIV integrase inhibitors. Other activities, such as antifungal and anticancer abilities, have also been reported [35].

The obtained results lead us to the conclusion that research on SQI compounds should continue both in the direction of modifying the styrene group in the molecular structure, as well as in terms of varying the doses of gamma radiation.

From a clinical perspective, due to the impossibility of experimentally irradiating people with varying doses, clinical studies of experimental animals deserve special attention. The studied compounds that exhibit a radiation protection effect could be used for the development of therapies for small animals by applying irradiation in a controlled manner to treat cancer, with the possibility of achieving improved quality of life and longer life expectancy.

## 4. Conclusions

The present study investigated the radioprotective effect of four newly synthesized organic compounds. According to the experimental results 3,5-dichloro-2-hydroxyphenylmethylene amino-benzoic acid in EtOH at a dose of 60 mg/kg body weight exhibited a radioprotective effect (25% survival after 30 days) against 8 Gy radiation of male Wistar rats. The 2-N-methyl-2-styrylquinoline salt manifested a lower survival rate (10%) up to the 15th day after irradiation. The alkaline–water solution of the herbal compound silymarin did not display radioprotective potential, even in doses surpassing the therapeutic does by 5–20 times. A comparative estimation of the experimental data revealed that the type of the solvent, especially for natural flavonoids, has a significant effect on the radioprotective activity of biologically active compounds. The obtained results highlight the necessity of future investigations on the radioprotective potential of the novel synthetic compounds, their derivatives, and the plant extract silymarin at various gamma radiation and injection doses.

## Figures and Tables

**Table 1:** In vivo radioprotective activity (expressed as survival percentage) of AZM1, AZM2, AZM2*, SQI, and silymarin.

Group (Number of Rats)	Compound	Solvent	Injection Dose, mg/kg	Irradiation Dose, Gy	Survival, %		
					10th Day	15th Day	30th Day
G1 (12)	AZM1	EtOH	60	8	0	0	0
G2 (8)	AZM2	EtOH	60	8	50	25	25
G3 (10)	AZM2	EtOH	200	8	10	0	0
G4 (9)	AZM2	DMSO	60	8	100	11	11
G5 (10)	AZM2*	H_2_O	60	8	0	0	0
G6 (10)	AZM2*	H_2_O	200	8	0	0	0
G7 (10)	SQI	H_2_O	0.3	8	60	10	0
G8 (10)	SQI	H_2_O	1.8	8	0	0	0
G9 (10)	silymarin	aqueous-alkaline medium (pH = 10)	40	8	0	0	0
G10 (10)	silymarin	aqueous-alkaline medium (pH = 10)	160	8	0	0	0
G11 (10) (control 1)	-	DMSO	8000	8	0	0	0
G12 (10) (control 2)	-	-	-	8	0	0	0
G13 (10) (control 3)	-	-	-	-	100	100	100

**Table 2:** Comparative analyses of scientific literature data on the in vivo radioprotective effect of silymarin.

Animals	Gamma Radiation Dose, Gy	Dose Rate	Silymarin Injection Dose, mg/kg	Solvent	Exposure Period	Results	Ref.
male albino rats, Rattus rattus, (130 ± 5 g)	4	0.713	70 mg/kg	H_2_O	21 days post radiation	Protection against gamma radiation injury	[20]
male albino Sprague–Dawley rats, (10 ± 2 weeks old; 120 ± 20 g)	6	0.43	50	H_2_O	Balanced diet for 8 weeks and exposed to γ-radiation (6 Gy) on the 4th week	Effective in the protection of liver damage induced by γ-radiation exposure	[21]
adult female albino rats (150 ± 50 g)	1 and 6	0.014	100	H_2_O	Twice daily for 1 week; last injection 2 h before blood sampling and 2 h pre-irradiation	Amelioration of radiation-induced blood disorders	[22]
male rats	6	0.2 and 0.6 Gy/day	100 mg/kg per day 70 mg/kg twice per day	NA		Positive effect of continual irradiation, with increase in mitotic activity and mitigation of chromosomal erration frequency in the regenerating liver	[23]
male albino rats	6	0.64 Gy/min	8 mM/kg	NA	10 days	Reduced alterations of the blood levels of LDH, CPK, MDA, GSH, GSH-Px, and SOD	[24]
adult male Wistar rats (180–220 g)	7	0.38 Gy/min	50 mg/kg	NA	Daily for 2 weeks	Modulating effect of choline glycerophosphate and silymarin against detrimental effects of gamma radiation in rats via the cholinergic anti-inflammatory pathway	[25]
female albino Wistar rats (150 ± 20 g)	4 Gy twice in a 4-day interval	0.43 Gy/min	100 mg/kg extract daily oil fraction, 0.5 mL/kg b. wt. daily	NA	12 consecutive days; 12 consecutive days	- Protective effect against γ-radiation-induced cell damage; - inhibited lipid peroxidation process in heart and brain tissues - Induced the production of antioxidant agents (GSH and MT) in the cells of rats exposed to γ-radiation	[26]
adult male mice (36–41 g)	2 Gy	0.5 Gy/min	50 mg/kg	NA	7 consecutive days	Prevente stress oxidative and inflammatory activity and enhanced the antioxidant defense system and regeneration of sperm cells	[27]
male Wistar rats (110–120 g)	8 Gy	0.02 Gy/s	40 and 160 mg/kg	aqueous-alkaline medium (pH = 10)	10, 15, and 30 days	100% rat mortality	Present study

**Table 3:** Energy of HOMO/LUMO orbitals in the molecules of AZM2 and AZM2*.

Molecular Descriptor	AZM2	AZM2*
HOMO energy, eV	−9.977	−9.009
LUMO energy, eV	−9.440	−3.598
HOMO-LUMO energy gap	0.537	5.411

## Data Availability

Not applicable.
